# Neurosonology in acute stroke revealing bilateral internal carotid artery occlusion maintained by persistent embryologic collaterals: A case report

**DOI:** 10.1016/j.radcr.2026.03.026

**Published:** 2026-04-18

**Authors:** Omar Ghomari Khayat, Leila Bentamra, Islem Houari, Rachid Belfkih, Didier Smadja, Nicolas Chausson

**Affiliations:** a Department of Neurology, Centre Hospitalier Sud-Francilien, Corbeil-Essonnes, France; b Department of Neurology, University Hospital Center Mohammed VI of Tangier, Tangier, Morocco; c Abdelmalek Essaadi University, Faculty of Medicine and Pharmacy of Tangier, Tangier, Morocco; d Institute of Psychiatry and Neuroscience of Paris (IPNP), INSERM U1266, Paris, France

**Keywords:** Neurosonology, Internal carotid artery occlusion, Persistent embryologic anastomoses, Duplex ultrasound, Collateral circulation

## Abstract

Neurosonology has become a pivotal modality in acute and subacute neurovascular assessment, providing rapid, non-invasive evaluation of arterial patency and cerebral hemodynamics. We report the case of a 49-year-old woman, active smoker, who presented with transient dysarthria and right brachio-facial weakness, leaving only mild residual dysarthria on admission. Brain MRI demonstrated a superficial infarction within the left middle cerebral artery territory. Extracranial duplex ultrasound played a decisive diagnostic role by revealing bilateral internal carotid artery occlusion and suggesting the presence of compensatory collateral circulation. These findings were subsequently corroborated by vascular imaging. The patient was managed with antithrombotic therapy, with favorable radiological evolution characterized by reperfusion of the previously occluded carotid axis. This observation underscores the fundamental contribution of neurosonology not only for early identification of major arterial occlusion, but also for functional assessment of flow dynamics and collateral supply. It reinforces duplex ultrasound as an indispensable component of comprehensive neurovascular work-up and a powerful complement to advanced angiographic modalities.

## Introduction

Internal carotid artery (ICA) stenosis and occlusion remain major contributors to ischemic stroke and carry a substantial risk of recurrence. Chronic symptomatic ICA occlusion is associated with an annual ipsilateral stroke/ transient ischemic attack risk of ∼ 7%, which may rise to nearly 24% within 2 years in patients with poor collateral compensation despite medical therapy. These figures highlight that prognosis depends not only on anatomical obstruction but critically on the adequacy of cerebral hemodynamics and collateral flow [1].

In this setting, neurosonology has become a pivotal imaging modality in stroke medicine. Modern cervical and transcranial ultrasound techniques enable rapid, bedside, non-invasive assessment of arterial patency, stenosis severity, flow direction, and collateral recruitment, providing real-time functional insight that complements CT and magnetic resonance angiography. Its ability to detect hemodynamic compromise, monitor perfusion, and guide clinical decision-making has firmly positioned neurosonology at the core of contemporary neurovascular evaluation [2].

Among collateral mechanisms, persistent embryologic carotid–vertebobasilar anastomoses are rare but clinically relevant variants. Although often incidental, they may become crucial alternative perfusion routes in the presence of severe carotid disease. Some of these arteries have reported incidences below 0.1%, yet can be directly implicated in maintaining posterior or anterior circulation when conventional vascular pathways fail, making their recognition essential for accurate interpretation and safe management [3].

Herein, we report a case of bilateral ICA occlusion in which neurosonology played a decisive diagnostic and hemodynamic role, while embryologic collateral pathways contributed to maintaining cerebral perfusion.

## Case presentation

A 49-year-old woman with a medical history of hysterectomy (2018) and active tobacco use (40 pack-years) presented to the Emergency Department with sudden-onset dysarthria associated with right brachio-facial weakness. Symptoms partially regressed spontaneously within a few hours. On admission, neurological examination revealed only a mild residual dysarthria, with a National Institutes of Health Stroke Scale (NIHSS) score of 1. There were no swallowing disturbances, cranial nerve palsy, sensory deficits, or lower limb weakness. Blood pressure, cardiac rhythm, and metabolic parameters were unremarkable. No prior stroke or cardiovascular disease was documented.

**Fig. 1:**
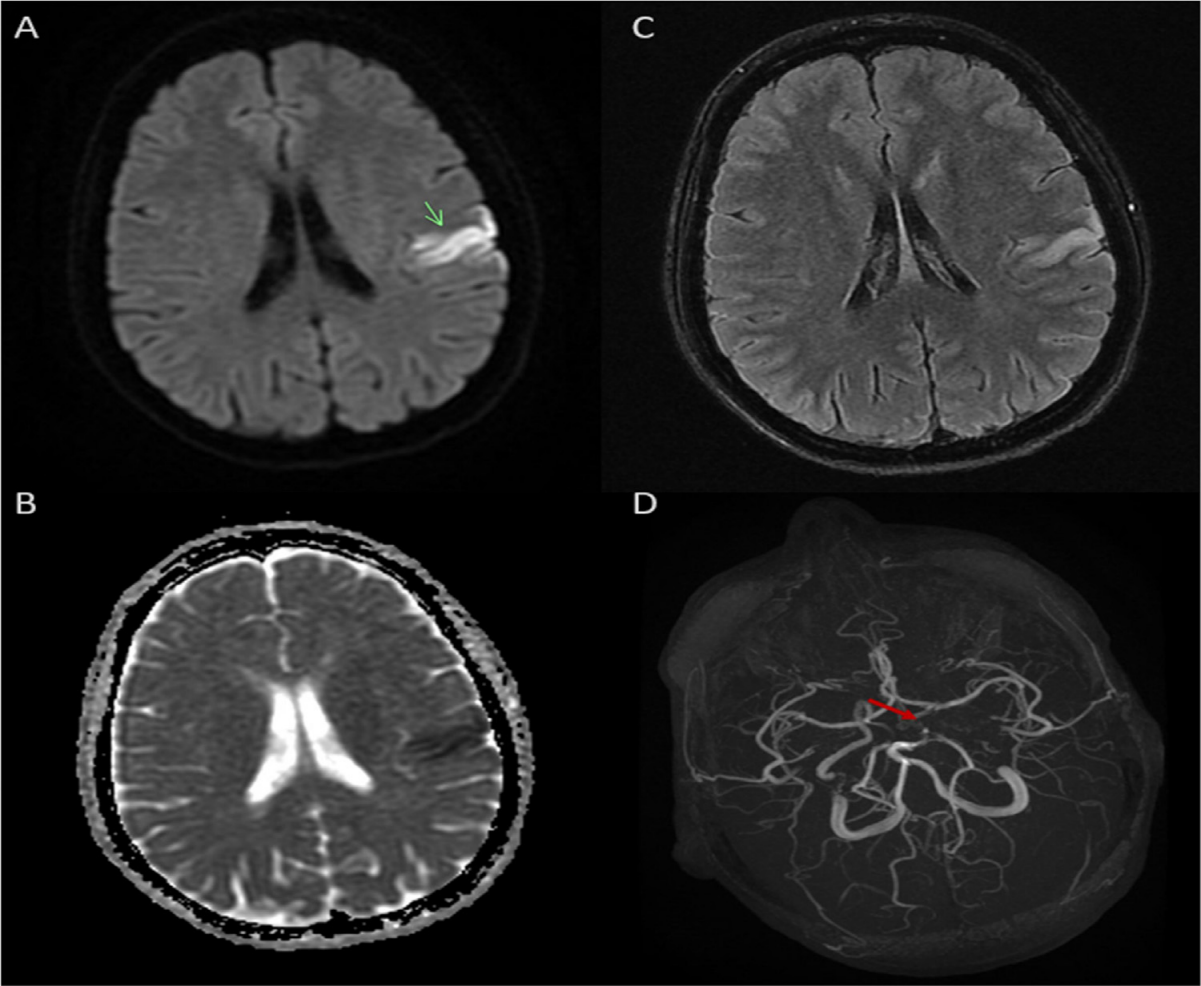
Brain MRI at presentation. (A) Axial DWI shows a focal cortical hyperintense lesion in the left fronto-parietal cortical region within the territory of a distal cortical branch of the middle cerebral artery MCA (green arrow). (B) ADC map demonstrates corresponding hypointensity, confirming true diffusion restriction. (C) Axial FLAIR sequence shows subtle matching cortical hyperintensity without mass effect or hemorrhagic transformation. (D) TOF-MRA reveals asymmetry of the anterior circulation, with reduced opacification and caliber of the left carotid/MCA inflow and irregular signal at the terminal ICA–A1/M1 junction (red arrow), suggesting hemodynamic compromise of the left ICA pathway with collateral compensation.

Brain MRI confirmed an acute ischemic lesion in the left fronto-parietal cortical region within the territory of a distal cortical branch of the middle cerebral artery (MCA), with focal diffusion restriction on diffusion-weighted imaging (DWI) and corresponding apparent diffusion coefficient (ADC) hypointensity. Fluid-attenuated inversion recovery (FLAIR) imaging showed subtle cortical hyperintensity without significant edema or mass effect. Susceptibility-weighted angiography (SWAN) sequences revealed no intraluminal thrombus or hemorrhagic transformation. Intracranial time-of-flight magnetic resonance angiography (TOF-MRA) demonstrated asymmetry of the anterior circulation, with reduced opacification of the left carotid inflow and less robust visualization of the left MCA trunk, suggesting hemodynamic compromise of the left ICA pathway with collateral compensation rather than a normal symmetric intracranial arterial tree (Fig. 1).

**Fig. 2:**
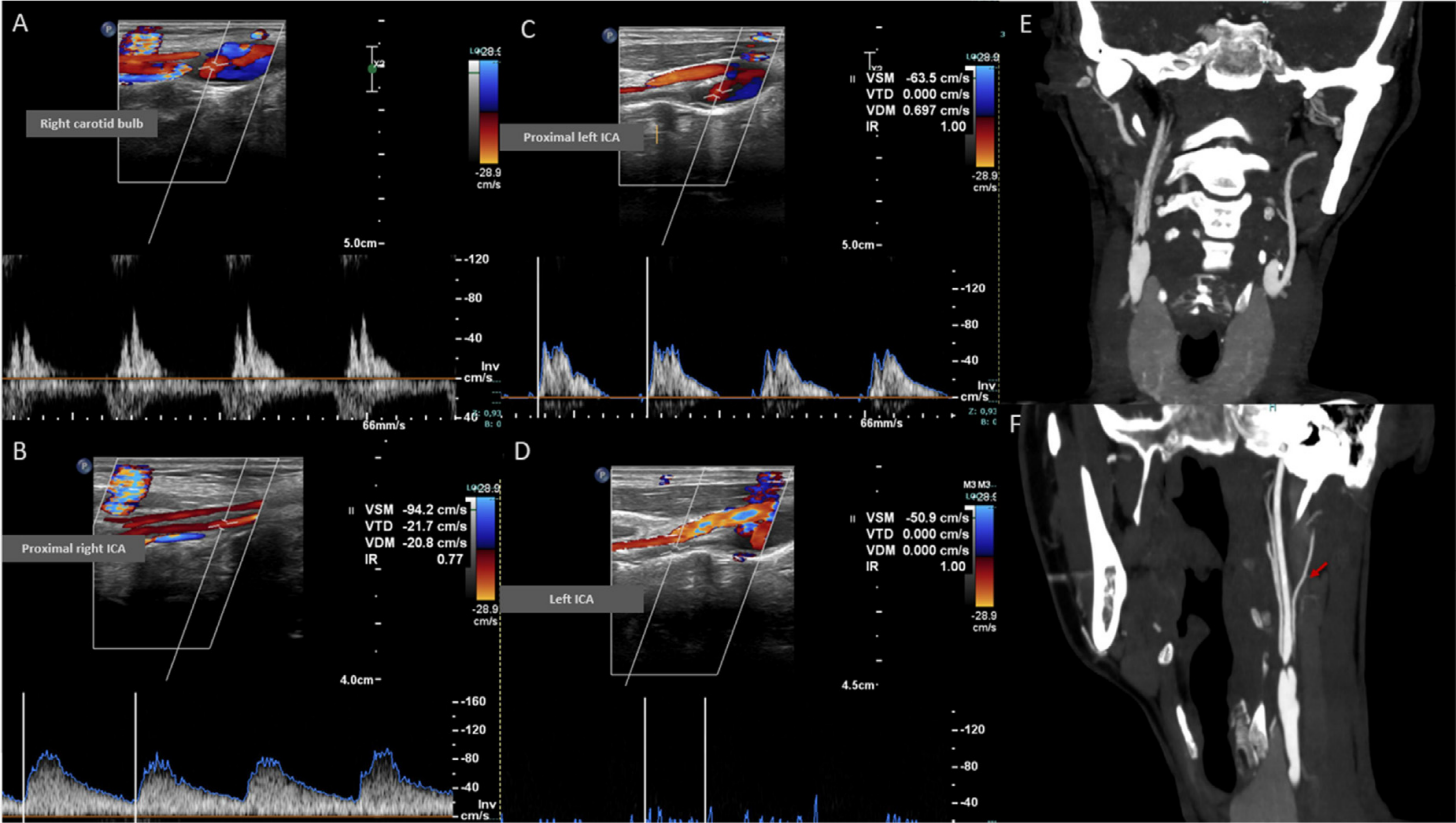
Cervical neurosonology and CTA showing bilateral ICA occlusion with asymmetric collateral reperfusion. (A) Right carotid bulb: high-resistance waveform with absent diastolic flow, indicating distal ICA outflow obstruction. (B) Proximal right ICA: dampened but preserved antegrade flow, consistent with collateral-dependent patency. (C–D) Left ICA: markedly reduced systolic velocities with absent diastolic flow, showing near-occlusion and functional hemodynamic collapse. (E–F) Cervical CTA: bilateral cervical ICA occlusion; right ICA distally reperfused via a collateral artery, highly suggestive of a persistent embryologic carotid–vertebrobasilar anastomosis, while the left ICA remains critically narrowed without effective reconstitution.

Neurosonological evaluation was performed as part of the stroke work-up (Fig. 2). Cervical duplex ultrasound revealed that on the right side, the common carotid artery showed preserved systolic flow with peak systolic velocities around 70-90 cm/s and a reduced diastolic component, indicating distal resistance. Right carotid bulb Doppler demonstrates a high-resistance waveform with absent forward diastolic flow and peak systolic velocity around 70-80 cm/s, consistent with distal internal carotid outflow obstruction. The right internal carotid artery was patent, displaying a pulsatile but dampened waveform with proximal ICA peak systolic velocities around 90-100 cm/s, confirming maintained intraluminal flow rather than true occlusion. Color Doppler revealed a collateral vessel with a clear retrograde flow direction anastomosing with the right ICA, supplying it in reverse fashion. Right ECA shows a typical high-resistance waveform with peak systolic velocity around 80-100 cm/s, consistent with a patent artery without hemodynamically significant stenosis.

On the left side, the common carotid artery exhibited a markedly high-resistance waveform with peak systolic velocities of approximately 70-85 cm/s with markedly reduced diastolic flow, consistent with downstream failure. The proximal ICA demonstrated severely reduced systolic activity and virtually absent diastolic flow, hemodynamically compatible with near-occlusion or functional occlusion. The left external carotid artery again showed increased velocities around 150-160 cm/s, indicating compensatory collateralization.

Cervical CT angiography demonstrates bilateral internal carotid artery occlusion at the cervical segment. The right internal carotid artery is proximally occluded but remains perfused through retrograde collateral inflow supplying the cervical ICA segment, indicating collateral reperfusion rather than true antegrade patency. The left internal carotid artery shows persistent non-opacification along its entire cervical course without distal reconstitution. Both common carotid arteries and external carotid arteries are well opacified. Intracranial arterial imaging further reveals markedly developed bilateral posterior communicating arteries, providing robust vertebro-carotid collateralization, along with prominent ophthalmic arteries, suggestive of external-to-internal carotid collateral pathways with likely retrograde flow, reflecting chronic hemodynamic adaptation to long-standing carotid insufficiency (Fig. 2).

**Fig. 3:**
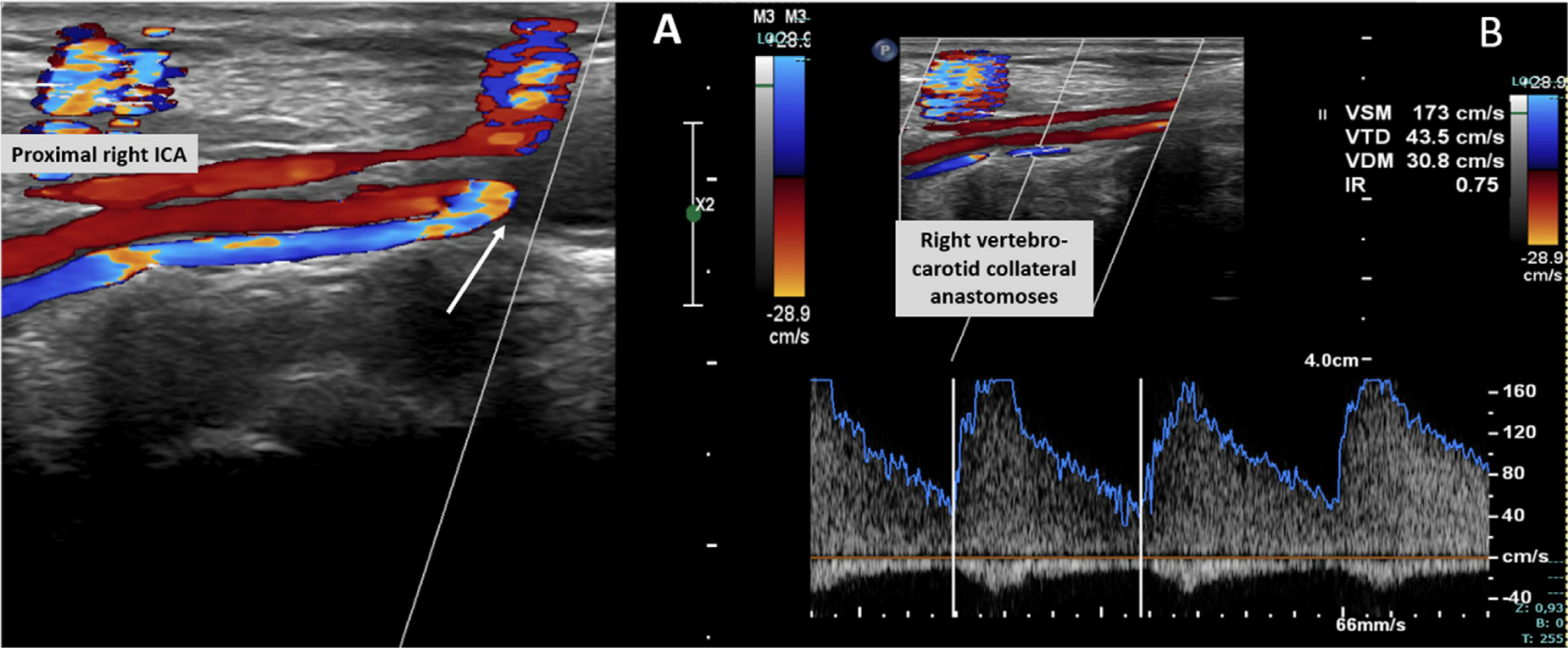
Duplex ultrasound of the right cervical carotid axis demonstrating proximal occlusion of the internal carotid artery with retrograde collateral inflow reconstituting the cervical ICA segment (white arrow) (A). A vertebro-carotid collateral vessel is identified; its direction and morphology are compatible with a persistent embryologic carotid–vertebrobasilar anastomosis, most likely a persistent hypoglossal artery or proatlantal intersegmental artery (white arrow). Spectral Doppler (B) shows high-velocity, low-resistance flow (PSV 173 cm/s, EDV 43.5 cm/s; RI 0.75), consistent with a hemodynamically significant collateral supplying the distal ICA territory.

Cervical duplex ultrasound and CTA demonstrated bilateral cervical ICA occlusion with asymmetric compensation. The right ICA is proximally occluded but reperfused via retrograde collateral inflow, while the left ICA remains functionally excluded without reconstitution, relying on ECA-derived collateralization. The morphology and flow direction of the right-sided collateral strongly suggest a persistent embryologic carotid–vertebrobasilar anastomosis, most likely a persistent hypoglossal artery or proatlantal artery (Fig. 3).

An extensive etiologic evaluation was performed to identify potential causes of bilateral cervical internal carotid artery occlusion. Cardiovascular assessment including transthoracic echocardiography and prolonged electrocardiographic monitoring did not reveal any cardioembolic source. Laboratory investigations showed no evidence of systemic inflammation or autoimmune disease, with negative testing for antinuclear antibodies, antineutrophil cytoplasmic antibodies, and antiphospholipid antibodies. Screening for thrombophilia, including protein C and protein S deficiency, antithrombin III deficiency, factor V Leiden mutation, and prothrombin gene mutation, was unremarkable.

Evaluation for non-atherosclerotic arteriopathies was also performed. Imaging studies did not demonstrate findings suggestive of fibromuscular dysplasia, arterial dissection, or vasculitis. In addition, there was no clinical or radiological evidence of Takayasu arteritis or other large-vessel inflammatory diseases. Metabolic and vascular risk factors, including lipid profile and glycemic status, were within normal limits.

Overall, the etiologic workup did not identify a definite cause for the bilateral carotid occlusion.

After confirmation of bilateral ICA occlusion with recent cortical infarction, antithrombotic therapy was initiated after multidisciplinary discussion, consisting of anticoagulation with dabigatran in combination with antiplatelet therapy using Aspirin, given the suspected thrombotic component. The therapeutic objective was to stabilize the thrombotic burden, prevent embolic recurrence, and potentially favor recanalization of the functionally occluded left ICA while preserving collateral hemodynamics. Blood pressure and lipid profile were optimized, smoking cessation was strongly advised, and no endovascular procedure was attempted in the acute phase given the chronic appearance of the cervical lesions and the preserved clinical status.

**Fig. 4:**
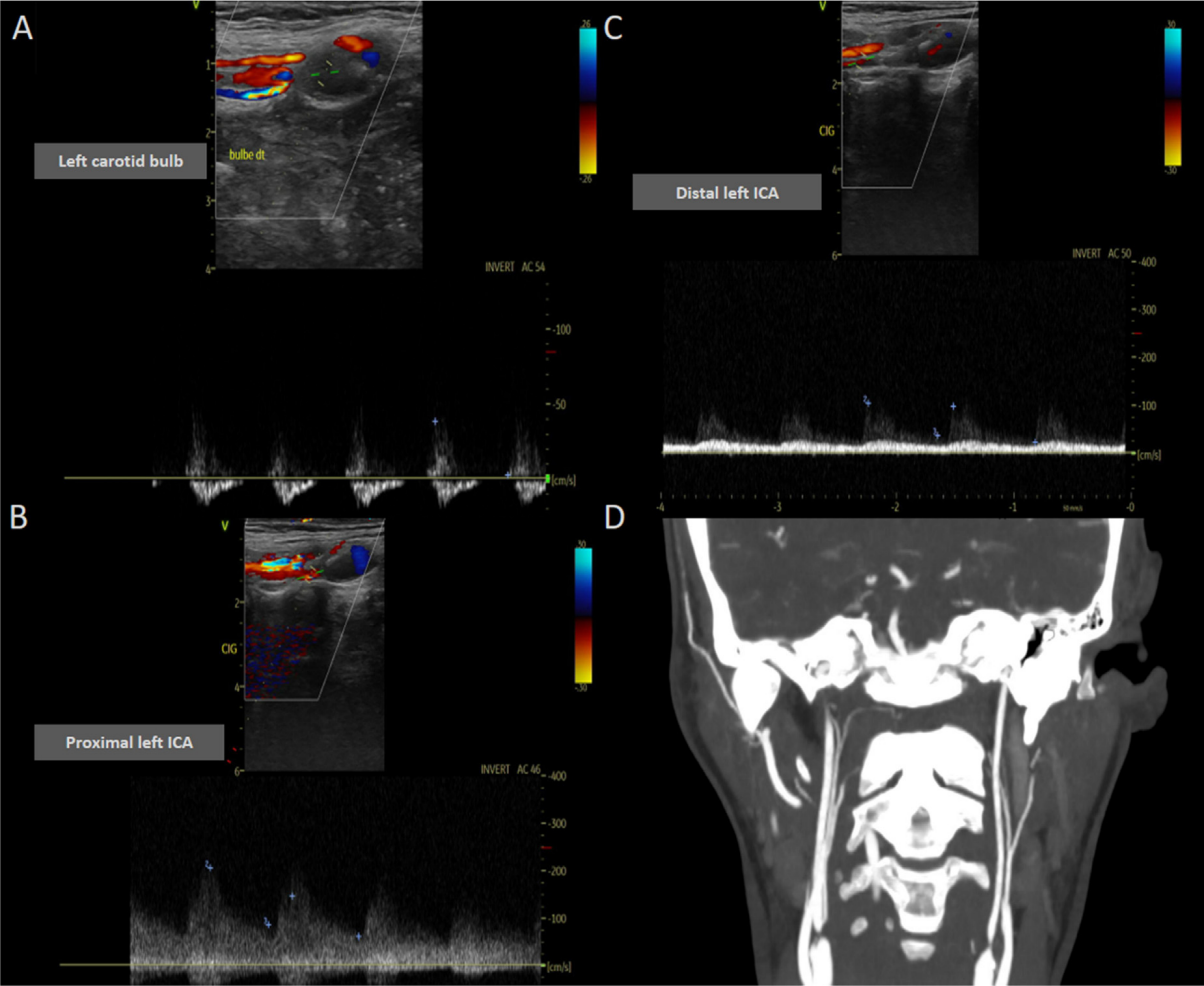
Follow-up neurosonology and cervical CTA demonstrating partial recanalization of the left ICA with persistent pre-occlusive hemodynamics. (A) Left carotid bulb Doppler shows reappearance of luminal flow but marked distal resistance, with low systolic velocity (PSV 38.1 cm/s) and near-absent diastole (EDV 2.3 cm/s; RI 0.94). (B) Proximal left ICA demonstrates high systolic velocity (PSV 190 cm/s) with preserved diastolic component (EDV 60.9 cm/s; RI 0.58), consistent with critical stenosis and turbulent reperfusion flow. (C) Distal left ICA shows persistent hemodynamic impairment with reduced systolic velocity (PSV 96.6 cm/s) and diminished diastolic flow (EDV 22.3 cm/s; RI 0.77), indicating incomplete normalization of downstream perfusion. (D) Coronal cervical CTA confirms thread-like recanalization of the left ICA rather than full-caliber restoration, supporting a pre-occlusive, critically narrowed reperfused state rather than true anatomical patency.

One month later, cervical duplex ultrasound and repeated cervical CTA documented partial reperfusion of the left ICA but not a return to normal physiology. Duplex ultrasound of the left carotid axis demonstrated marked hemodynamic disturbance. At the carotid bulb, luminal flow reappeared but with pronounced distal resistance, characterized by low systolic velocity (PSV 38.1 cm/s) and near-absent diastolic flow (EDV 2.3 cm/s; RI 0.94), consistent with downstream obstruction. The proximal internal carotid artery showed markedly elevated systolic velocity (PSV 190 cm/s) with preserved diastolic component (EDV 60.9 cm/s; RI 0.58), reflecting turbulent flow associated with proximal occlusion and collateral reperfusion. Distally, the ICA exhibited persistent hemodynamic impairment with reduced systolic velocity (PSV 96.6 cm/s) and diminished diastolic flow (EDV 22.3 cm/s; RI 0.77), indicating incomplete normalization of downstream perfusion. This pattern is concordant with cervical CT angiography, which shows a narrow, thread-like recanalized segment of the left ICA rather than a widely opacified lumen (Fig. 4).

The patient remained clinically stable with no residual neurological deficit, reflecting good functional recovery. The patient was initially treated with dual antithrombotic therapy. After one month, treatment was simplified to Aspirin monotherapy for long-term secondary stroke prevention. Follow-up cervical duplex ultrasound and CT angiography demonstrated stable vascular findings, with persistent bilateral internal carotid artery occlusion and maintained collateral perfusion, without evidence of progression or new ischemic lesions.

In summary, this case illustrates bilateral cervical internal carotid artery occlusion with markedly asymmetric hemodynamic adaptation. Duplex ultrasonography demonstrated that the right ICA, although proximally occluded, remained functionally perfused through retrograde collateral inflow into the cervical segment, suggesting a vertebro-carotid collateral pathway compatible with a persistent embryologic carotid–vertebrobasilar anastomosis. In contrast, the left ICA evolved from complete occlusion to a critically stenotic pre-occlusive state with partial recanalization under dual antithrombotic therapy. This case highlights the diagnostic value of neurosonology in distinguishing true arterial occlusion from collateral-maintained patency, identifying atypical collateralization patterns, and monitoring the dynamic hemodynamic evolution of cervical carotid lesions on follow-up.

## Discussion

Management of internal carotid artery occlusion (ICAO) remains primarily conservative, built on antiplatelet therapy, high-intensity statins, and strict vascular risk factor control. Contemporary cohorts suggest that medically treated symptomatic ICAO still carries an annual ipsilateral stroke risk of roughly 5%-7%, rising above 20% at two years in patients with hemodynamic compromise [1]. Surgical EC–IC bypass failed to improve outcomes in randomized trials: the Carotid Occlusion Surgery Study (COSS) showed no reduction in 2-year ipsilateral stroke with STA–MCA bypass plus best medical therapy versus best medical therapy alone (21.0% vs 22.7%), while introducing a ≈ 14%-15% peri-operative stroke risk [4]. As a result, bypass is now reserved, if used at all, for highly selected patients with proven hemodynamic failure.

In contrast, endovascular recanalization of chronic ICAO has gained momentum. A 2020 meta-analysis of 13 studies (528 patients) reported successful recanalization in about 70% of cases with ≈ 5% procedure-related morbidity and a marked reduction (≈80%) in subsequent thromboembolic events compared with medical therapy alone [5]. A larger 2024 meta-analysis of carotid occlusion endovascular surgery confirmed high technical success and low rates of periprocedural ischemic and hemorrhagic complications, supporting endovascular recanalization as an effective and reasonably safe option in carefully selected patients [1]. Recent series and reviews emphasize that outcomes depend heavily on occlusion morphology and chronicity, and that hybrid carotid endarterectomy–endovascular strategies can further improve recanalization rates in long-segment or cavernous-segment occlusions [6]. Overall, current literature supports a stratified approach: best medical therapy as the foundation, with endovascular or hybrid revascularization reserved for symptomatic, hemodynamically compromised ICAO after multidisciplinary evaluation.

Persistent carotid–vertebrobasilar anastomoses (PCVBA) are rare remnants of fetal arteries that connect the carotid and vertebrobasilar systems—classically the persistent trigeminal, hypoglossal, and proatlantal intersegmental arteries. They result from failure of regression of these primitive channels when the vertebral and posterior communicating arteries develop [3]. The persistent trigeminal artery (PTA) is the most frequent, with an estimated prevalence of 0.1%-0.6%, whereas the persistent hypoglossal artery (PHA) is the second most common, reported in 0.02%-0.26% of angiographic studies; persistent proatlantal arteries (type I and II) are exceptionally rare [7]. These variants are often associated with hypoplastic or absent vertebral and/or posterior communicating arteries, so that a single embryologic vessel may become the dominant supply to the posterior circulation [8].

Clinically, most PCVBA are discovered incidentally on CTA/MRA or catheter angiography, but their recognition is crucial in the context of stroke, aneurysm, and endovascular procedures. Multiple case series report an increased prevalence of intracranial aneurysms—up to about 25%-30% in some PHA cohorts—as well as atypical stroke patterns due to unusual embolic pathways or hemodynamic stress at the carotid–basilar junction [9,10]. Persistent proatlantal arteries have been described in association with vertebral aplasia and complex malformations of the deep venous system, and can critically influence posterior circulation perfusion and the risk–benefit balance of carotid or vertebral interventions [11,12].

Recent work has specifically highlighted the role of neurosonology in detecting these embryologic collaterals. A 2025 ultrasound series of 35 patients with PCVBA showed that duplex can reliably identify PTA, PHA and proatlantal arteries, characterize flow direction, and document their compensatory role when the vertebral or ICA pathways are hypoplastic or occluded [13]. In this context, persistent vertebro–carotid anastomoses should be considered not only as anatomical curiosities, but as potentially critical collaterals or risk modifiers in patients with carotid occlusion, posterior circulation ischemia, or when planning neurointerventional or surgical procedures.

## Conclusion

This case illustrates an exceptional scenario of bilateral internal carotid artery occlusion with profoundly asymmetric compensatory hemodynamics, in which the right ICA remained functionally reperfused through a persistent embryologic carotid–vertebrobasilar anastomosis, while the left ICA evolved from complete occlusion to a pre-occlusive recanalized state under dual antithrombotic therapy. Brain MRI confirmed a limited superficial MCA infarction without hemorrhagic transformation, highlighting the protective role of collateral networks.

Overall, this observation emphasizes the dynamic nature of ICA “occlusion,” the decisive impact of persistent embryologic collaterals on cerebral perfusion, and reinforces the pivotal role of neurosonology combined with CTA/MRA in diagnosis, hemodynamic assessment, therapeutic orientation, and follow-up in complex cerebrovascular disease.

## Ethical approval

Ethics committee approval was not required for this single retrospective case report in accordance with institutional policy and the Declaration of Helsinki.

## Data availability

All relevant data are included in the article.

## Author contributions

All authors contributed to data acquisition, interpretation, and manuscript preparation and approved the final version.

## Patient consent

Written informed consent was obtained from the patient for publication of this case report and any accompanying images.
